# The extent to which standardized uptake values reflect FDG phosphorylation in the liver and spleen as functions of time after injection of ^18^F-fluorodeoxyglucose

**DOI:** 10.1186/s13550-017-0254-7

**Published:** 2017-02-07

**Authors:** Georgia Keramida, Constantinos D. Anagnostopoulos, A. Michael Peters

**Affiliations:** 1 Clinical Imaging Sciences Centre, Brighton Sussex Medical School, Brighton, UK; 2 Center for Experimental Surgery, Clinical and Translational Research, Biomedical Research Foundation Academy of Athens, Athens, Greece; 3 Department of Nuclear Medicine, Royal Sussex County Hospital, Eastern Road, Brighton, BN2 5BE UK

**Keywords:** FDG clearance, SUV, Liver, Spleen

## Abstract

**Purpose:**

In FDG PET/CT, standardized uptake value (SUV) is used to measure metabolic activity but detects un-phosphorylated FDG as well as phosphorylated FDG (FDG6P). Our aim was to determine the proportions of intrahepatic and intrasplenic FDG that are phosphorylated after FDG injection and compare them with SUVs.

**Methods:**

Sixty patients undergoing whole-body PET/CT 60 min post-injection of FDG first had dynamic PET imaging for 30 min with measurement of hepatic and splenic FDG clearances using Patlak-Rutland analysis. The gradient of the Patlak-Rutland plot, which is proportional to clearance (Ki), was normalized to the intercept, which is proportional to FDG distribution volume (*V*(0)) with the same proportionality constant. Using measured values of Ki/*V*(0), FDG6P/FDG ratios as functions of time in the two organs were measured for assumed FDG blood disappearance half-times of 40, 50 and 60 min. Hepatic and splenic SUVs were measured from whole-body PET/CT.

**Results:**

The mean (SD) Ki/*V*(0) was 0.0036 (0.0021) and 0.0060 (0.0041) ml/min/ml for the liver and spleen, respectively, but the hepatic SUV was 1.36-fold higher than the splenic SUV. This discrepancy was explained by the hepatic *V*(0) being 1.6-fold higher than the splenic *V*(0). The percentages of FDG phosphorylated 60 min post-injection were 27, 25 and 23% for the liver and 39, 36 and 34% for the spleen, for blood clearance half-times of 40, 50 and 60 min, respectively. SUV indices correlated poorly with Ki/*V*(0) for both organs.

**Conclusions:**

SUV is largely determined by un-phosphorylated FDG in dynamic exchange with blood FDG, explaining the poor correlations between SUV indices and Ki/*V*(0).

## Background

**Fig. 1:**
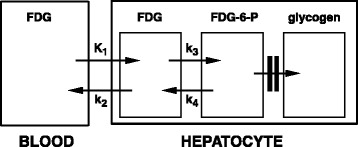
Model of intrahepatic FDG kinetics. *K*_1_ is hepatic blood flow, *k*_2_ is a diffusion constant, *k*_3_ the glucokinase and k_4_ glucose-6-phosphatase. FDG is assumed to mix throughout its intrahepatic distribution volume via *K*_1_ and *k*_2_ by 2–3 min post-injection. De-phosphorylation (via *k*_4_) is assumed to be slow enough to ignore in Patlak-Rutland analysis, which therefore measures phosphorylation rate. The corresponding model for the spleen is similar except *k*_4_ is lacking

Positron emission tomography (PET) with 2-deoxy-2-[^18^F]fluoro-D-glucose (FDG) is widely used to measure tissue glucose utilization rate (MRglu). MRglu is the generation rate of glucose-6-phosphate via glucokinase-mediated phosphorylation. FDG, which is an analogue of glucose, also undergoes glucokinase-mediated phosphorylation to FDG-6-phosphate (FDG6P) (Fig. 1). Taking into account a ‘lumped constant’, introduced to account for differing tissue uptake kinetics between glucose and FDG (for example differing extraction efficiencies), MRglu (μmol/min/ml) is measured as the tissue FDG clearance rate (ml/min/ml) multiplied by blood glucose (μmol/ml). Tissue FDG uptake, however, is often quantified as the standardized uptake value (SUV) recorded on whole-body imaging, conventionally 60 min post-injection of FDG. SUV is defined as follows:

1 $$ \mathrm{S}\mathrm{U}\mathrm{V} = F/\mathrm{ml} \times \mathrm{whole}\ \mathrm{body}\ \mathrm{metric} $$

where *F*/ml is the fraction of administered activity per ml of tissue; the whole-body metric may be weight (SUW), lean body mass (LBM; SUL) or body surface area. Moreover, SUV may be based on the voxel with the maximum value (SUV_max_) or taken as the mean of all voxels in the region of interest (ROI; SUV_mean_).

In addition to the amount of FDG6P in a tissue, SUV also depends on the amount of un-phosphorylated FDG, which is free to exchange between tissue and blood [1–4].

Like FDG clearance, SUV is inversely related to blood glucose in insulin-insensitive tissues such as tumours [5] and brain [6, 7]. SUV is therefore a surrogate of FDG clearance rather than of MRglu and becomes a closer surrogate of MRglu after normalization to (i.e. multiplication by) blood glucose, as recommended in the European Association of Nuclear Medicine guidelines on PET/CT [8].

There have been several recent studies of FDG accumulation in the liver, mostly in relation to hepatic steatosis, that have used SUV as the quantitative index of FDG accumulation and measure of hepatic metabolic activity [9–13]. However, these studies have largely ignored the issue of how much intrahepatic FDG is actually phosphorylated. The same applies to the spleen, the metabolic activity of which, almost exclusively based on SUV in previous PET studies, has attracted a great deal of recent attention in relation to atherosclerosis [14] and acute myocardial infarction [15, 16], the so-called cardio-splenic axis. Using a combined theoretical and experimental approach, the aim of this study was to determine the proportions of FDG in the liver and spleen that are phosphorylated at specific post-injection times and the extent to which SUV reflects FDG clearance in these two organs.

## Methods

### Theory

The concentration of FDG6P in tissue at time *t* post-injection can be calculated from the tissue FDG clearance (Ki) and the area under the blood time-concentration curve up to time *t* (*A*(*t*)) as follows.

2 $$ \begin{array}{l}\mathrm{FDG}6\mathrm{P}\kern0.5em  = A(t) \times \mathrm{Ki}\\ {}\Big(\mathrm{MBq}/\mathrm{ml} = \left[\mathrm{MBq}/\mathrm{ml} \times \min \right] \times \mathrm{ml}/ \min /\mathrm{ml}\end{array} $$

The concentration of un-phosphorylated FDG (FDG) in tissue at time *t* post-injection can be calculated from the blood FDG concentration at time *t* (*C*(*t*)),

3 $$ \begin{array}{l}\mathrm{FDG} = C(t) \times V(0)\\ {}\left(\mathrm{MBq}/\mathrm{ml} = \mathrm{MBq}/\mathrm{ml} \times \mathrm{ml}/\mathrm{ml}\right)\end{array} $$

where *V*(0) is the volume of distribution of FDG in the tissue (i.e. the virtual volume within which FDG would be distributed to give an FDG concentration identical to plasma concentration). (If hepatic FDG concentration was identical to plasma concentration, then *V*(0) would be 1 ml/ml.)

Dividing Eq. 2 by Eq. 3

4 $$ \mathrm{F}\mathrm{D}\mathrm{G}6\mathrm{P}/\mathrm{F}\mathrm{D}\mathrm{G} = \left[ A(t)/ C(t)\right] \times \left[\mathrm{Ki}/ V(0)\right] $$

Note that *A*(*t*)/*C*(*t*) is normalised time in a Patlak-Rutland plot.

There is a very extensive literature on FDG arterial blood clearance data [1, 2, 17–28], but most publications aimed to validate simplifications of arterial input and very few to quantify the kinetics of blood FDG clearance. From an inspection of published data, the clearance curve can be seen to be a triple exponential with a very fast first exponential that is completed within seconds and a second exponential that is fast with a half-time of <5 min. The area enclosed by these early exponentials is small compared to the area under the slowest exponential and can be ignored. Three groups in particular have published clearance data in large patient numbers, and from their illustrations, the half-times of the slow exponentials can be seen to be about 40 min [23], 50 min [19] or 60 min [21, 22, 25].

Ignoring the fast exponentials, the area under the blood clearance curve is given as

5 $$ A(t) = \left( N/\beta \right)\ .\left.\left(1 - {\mathrm{e}}^{-\beta . t}\right)\right] $$

where *N* is the zero time intercept and *β* the rate constant of the slow exponential,

and

6 $$ C(t) = N.{\mathrm{e}}^{-\beta . t} $$

Therefore,

7 $$ A(t)/ C(t) = \left(1 - {\mathrm{e}}^{-\beta . t}\right)/\beta .{e}^{-\beta . t} $$

For blood disappearance half-times of 40, 50 and 60 min, *A*(*t*)/*C*(*t*) is 105, 94 and 87 min, respectively.

The total tissue concentration of FDG (FDG6P + FDG), which determines SUV, is given by Eqs. 2 and 3:

8 $$ \mathrm{F}\mathrm{D}\mathrm{G}6\mathrm{P} + \mathrm{F}\mathrm{D}\mathrm{G} = \left[ A(t).\ \mathrm{Ki}\right] + \left[ C(t). V(0)\right] $$

9 $$ \mathrm{F}\mathrm{D}\mathrm{G}6\mathrm{P} + \mathrm{F}\mathrm{D}\mathrm{G} = \left( V(0). A(t).\left[\mathrm{Ki}/ V(0)\right]\right) + \left[ C(t). V(0)\right] $$

It can be seen from Eq. 9 that the relationship between the total activity and Ki/*V*(0) has a gradient of *V*(0).*A*(*t*) and an intercept of *V*(0).*C*(*t*).

Combining Eqs. 5, 6 and 9

10 $$ \mathrm{F}\mathrm{D}\mathrm{G}6\mathrm{P} + \mathrm{F}\mathrm{D}\mathrm{G} = \left[ V(0).\left( N/\beta \right).\left(1 - {\mathrm{e}}^{-\beta . t}\right)\right].\ \mathrm{K}\mathrm{i}/ V(0) + \left[ V(0). N.{\mathrm{e}}^{-\beta . t}\right] $$

When *t* = infinity, it can be seen from Eq. 10 that

11 $$ \mathrm{F}\mathrm{D}\mathrm{G}6\mathrm{P} + \mathrm{F}\mathrm{D}\mathrm{G} = V(0).\left( N/\beta \right).\ \left(\mathrm{Ki}/ V(0)\right) $$

Therefore, when

12 $$ \mathrm{K}\mathrm{i}/ V(0) = \beta $$

13 $$ \mathrm{F}\mathrm{D}\mathrm{G}6\mathrm{P} + \mathrm{F}\mathrm{D}\mathrm{G} = V(0). N $$

That is, the total activity remains constant as a function of time following injection when Ki/*V*(0) = *β*.

### Patients

Sixty patients had dynamic PET prior to routine clinically indicated PET/CT. These patients formed the study group for a previous study that investigated the relationships of hepatic MRglu with hepatic steatosis and obesity [29]. Thirty-eight (including 12 with metabolically active lymphoma) had FDG-avid malignancy on routine PET/CT. Forty-seven were men (age range 28–84) and 13 were women (age 40–67). Nineteen patients had hepatic steatosis (CT density ≤40 HU on whole-body PET/CT), 18 were obese (BMI ≥30 kg/m^2^) and 5 had type 2 diabetes mellitus (none with type 1). Five patients had blood glucose >7 mmol/l, including one with >10 mmol/ml (three of these five were diabetic). Twelve patients had received chemotherapy within 6 months of their scan, 19 had received chemotherapy >6 months previously and 29 were chemotherapy-naïve. Patients with known or suspected high ethanol intake were excluded. All patients gave written informed consent for the study, which was approved by a local institutional review board (NRES Committee South Central - Oxford C: ref 13/SC/0231).

### Whole-body imaging and image analysis

Patients fasted for 6 h before FDG injection. Blood glucose was measured using a glucometer (ACCU-CHEK Performa; Inform ll strips; USA). Whole-body PET/CT was performed at 60 min post-injection of ~400 MBq FDG (not adjusted for body weight) using a Siemens Biograph 64-slice PET/CT scanner (Erlangen, Germany) with immediate non-enhanced CT scanning (120 Kvp/50 mA—Care dose 4D; slice 5 mm; pitch 0.8; rotational speed 0.5/s) for attenuation purposes only. 3D emission data was then acquired at 3 min per bed position (PET reconstruction: 4 iterations; subset 8; Gaussian pre-filter; FWHM 5 mm; matrix size 168 × 168; zoom 1).

Hepatic SUV indices and CT density were measured from a circular ROI of 3-cm diameter over the right lobe of the liver, and spleen SUV indices from a circular ROI of 2.5-cm diameter over the centre of the organ. Body weight and LBM, estimated from height and weight using the equations of Boer [30], were used as the whole-body metrics to give SUW and SUL, respectively. Both were expressed as the maximum voxel SUV (SUW_max_ and SUL_max_) and mean SUV in the ROI (SUW_mean_ and SUL_mean_). We assume that because it is soluble in water rather than fat, negligible FDG enters the fat droplets in hepatocytes. Using an equation relating fat content to CT density [31], hepatic SUV was therefore adjusted for the physical ‘dilutional’ effect of hepatic fat on the FDG signal, as previously described [32].

### Dynamic imaging and image analysis

Prior to whole-body imaging, dynamic imaging was performed as 30 × 1-min frames following FDG injection with detectors positioned over the torso. Hepatic and splenic FDG clearances were measured using Patlak-Rutland graphical analysis from ROI over the liver (3 cm) and spleen (2.5 cm), each summed from 20 contiguous cranio-caudal transaxial slices, avoiding any suspected focal pathology in each slice, as previously described [29]. All frames were corrected for physical decay of ^18^F. Input function was derived from ROI over the abdominal aorta, within and avoiding the walls, summed from about 20 contiguous cranio-caudal transaxial slices drawn by a single operator (GK). The gradient of the plot, which represents tissue FDG clearance (Ki), was divided by the intercept, which represents tissue distribution volume of FDG (V(0)—see Eq. 3). There was no need for attenuation correction of the dynamic study because the factors that respectively relate gradient and intercept based on raw count rates to Ki and *V*(0) are identical, and cancel out in their ratio, as previously proven [29].

**Fig. 2:**
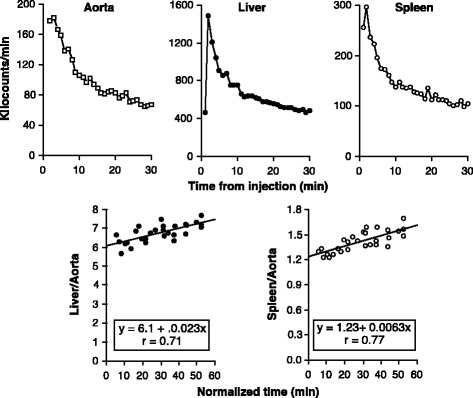
Examples of raw data and Patlak-Rutland plots for the liver and spleen in the same patient. *Upper panels*: abdominal aorta (*left*); liver (*middle*) and spleen (*right*). *Lower panels*: Patlak-Rutland plots for the liver (*left*) and spleen (*right*). Note that for both organs, the first two frame points have been excluded

We did not measure the transport constants governing tissue FDG kinetics shown in Fig. 1. Previous workers have shown that *K*_1_ (which represents tissue blood flow) and *k*_2_ (the constant of diffusion of un-phosphorylated FDG from tissue to blood) are sufficiently high with respect to the liver that FDG mixes throughout its hepatic distribution volume within 2–3 min. The first two frames of the dynamic study (0–1 min and 1–2 min) over the liver were therefore excluded from the Patlak-Rutland plot to allow mixing of the FDG throughout its hepatic distribution volume. The validity of this is apparent from Fig. 2, which shows the Patlak-Rutland plot to be essentially linear from the third frame. Although the corresponding transport constants for the spleen have not, to our knowledge, been determined, the plots for the spleen were also linear from the third frame, suggesting that the mixing time of FDG in splenic *V*(0) is as rapid as in liver *V*(0). Linearity of the plots also suggests that de-phosphorylation of FDG6P is very slow. Otherwise, the plot would be convex upwards. De-phosporylation takes place via glucose-6-phosphatase (*k*_4_), which is absent from the spleen. The essential requirement of the Patlak-Rutland plot for a single transport constant is therefore met for both tissues when the plot is started from 3 min.

The ratio of FDG6P to total tissue FDG concentration (FDG6P/[FDG6P + FDG]) was calculated from Eqs. 4 and 7 using *A*(*t*)/*C*(*t*) based on slow exponential half-times of 40, 50 and 60 min (corresponding to *β* of 0.0173, 0.0139 and 0.0115 min^−1^, respectively). Total ^18^F tracer concentrations were estimated using Eq. 9. *C*(*t*) and *V*(0) are unknown in Eq. 9, so the absolute concentration of tracer is unknown. However, the time courses of tissue FDG6P + FDG concentration for different values of Ki/V(0) can be derived because *V*(0) is independent of post-injection time.

### Statistics

Using the Shapiro-Wilk test, the *W* statistic gave *p* > 0.05 for all SUV indices and Ki/*V*(0), so parametric statistics were used. Values were expressed as mean ± standard deviation (SD). Pearson’s correlation analysis was used to assess relationships between SUV indices and Ki/*V*(0).

## Results

### Ki/*V*(0)

Representative examples of hepatic and splenic Patlak-Rutland plots from the same patient are shown in Fig. 2. Both Patlak-Rutland plots are essentially linear from the third frame. For all 60 patients, the mean (SD) Ki/*V*(0) of the liver was 0.0036 (0.0021) and of the spleen was 0.0060 (0.0041) ml/min/ml (*p* < 0.0001).

### Estimations of phosphorylated (FDG6P) and un-phosphorylated FDG

**Fig. 3:**
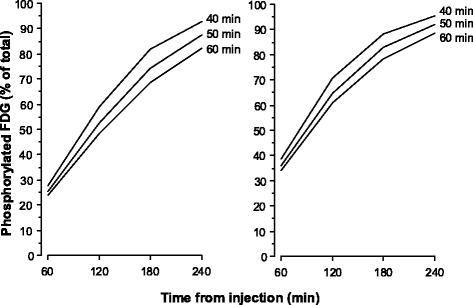
Percentage of tissue FDG that is phosphorylated as a function of time after FDG injection shown for slow exponential half-times of 40, 50 and 60 min; *left panel*: Ki/*V*(0) = 0.0036 ml/min/ml (typical for the liver); *right panel*: Ki/*V*(0) = 0.006 ml/min/ml (typical for the spleen)

Using the above mean values of Ki/*V*(0), the percentages of FDG phosphorylated 60 min post-injection for blood clearance half-times of 40, 50 and 60 min were calculated to be 28, 25, and 23% for the liver and 39, 36 and 34% for the spleen (Fig. 3). In either organ, percentages approaching 100% would not be achieved until about 4 h post-injection.

**Fig. 4:**
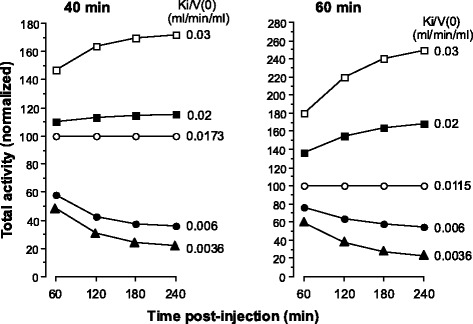
Time courses of total tissue activity estimated using Eq. 9 for slow exponential half-times of 40 min (*left panel*) and 60 min (*right panel*). *V*0 has been set to unity. The vertical axis is the total tissue activity (FDG6P + FDG) normalized to 100. When *t* is infinity, *C*_*t*_ = 0 and *A*_*t*_ = *N*/*β* (Eqs. 5 and 6). Therefore, when Ki/*V*(0) = *β*, the total activity is constant over post-injection time. For a slow exponential half-time of 40 min, *β* = 0.0173 min^−1^. Therefore, for values of Ki/*V*(0) > 0.0173 ml/min/ml, total activity increases as a function of post-injection time, and vice versa when Ki/*V*(0) < 0.0173 ml/min/ml. The boundary Ki/*V*(0) value for a slow exponential half-time of 60 min is 0.0115 ml/min/ml. Ignoring the small areas under the fast exponentials does not alter these time courses

Based on a slow exponential half-time of 40 min, it can be seen from Fig. 4 that total tissue concentration (FDG6P + FDG) increases or decreases as a function of post-injection time for values of Ki/*V*(0) respectively greater than or less than *β* (0.0173 ml/min/ml). For slow exponential half-times of 50 and 60 min, *β* is 0.0139 and 0.0115 min^−1^, so the boundary Ki/*V*(0) values are 0.0139 and 0.0115 ml/min/ml, respectively.

### SUV indices at 60 min post-injection

**Fig. 5:**
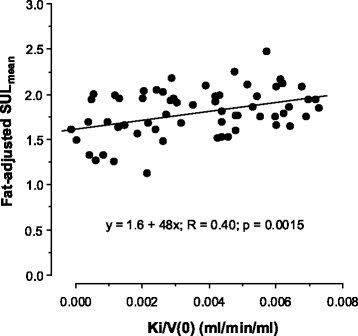
Relationship between fat-adjusted hepatic SUL_mean_ and Ki/*V*(0) (see Table 1). Note the large positive zero time intercept, consistent with Eq. 9

Hepatic SUV was higher than splenic SUV. Thus, the liver-to-spleen SUV_mean_ and SUV_max_ ratios (which, as ratios, are independent of whole-body metric) were 1.23 (0.24) and 1.36 (0.26), respectively (*p* < 0.0001 versus 1 for both). SUV indices generally correlated weakly with Ki/*V*(0) with respect to both the liver and spleen (Table 1). With respect to the liver, but not the spleen, SUL indices correlated more strongly with Ki/*V*(0) than SUW indices, and SUV_max_ indices correlated more strongly than SUV_mean_ indices. Adjusting the liver SUV_mean_ indices for hepatic fat improved their correlations with Ki/*V*(0). Relationships of SUV indices with Ki/V(0) had relatively large intercepts (Fig. 5), which, because SUV reflects the total tissue concentration, is consistent with Eq. 9. With respect to the spleen, division of SUV indices by hepatic SUV_mean_ improved their correlations with Ki/V(0) but no differences were seen between SUV_max_ and SUV_mean_ indices.

**Table 1:** Correlation coefficients (*p*) of relationships of hepatic and splenic SUV indices with hepatic and splenic Ki/*V*(0)

	Liver	Spleen
SUW_mean_	0.17 (0.36)	0.24 (0.065)
SUW_max_	0.29 (0.045)	0.24 (0.065)
SUL_mean_	0.20 (0.17)	0.25 (0.054)
SUL_max_	0.38 (0.005)	0.26 (0.045)
FA SUW_mean_	0.31 (0.030)	–
FA SUL_mean_	0.40 (0.0015)	–
SUV_mean_/hepatic SUV_mean_	–	0.40 (0.0015)
SUV_max_/hepatic SUV_mean_	–	0.40 (0.0015)

## Discussion

We found that at 60 min post-injection, only about 25% of FDG is phosphorylated in tissues, such as the liver, with Ki/V(0) of 0.0036 ml/min/ml, and about 35% in tissues, such as the spleen, with Ki/V(0) of 0.006 ml/min/ml. The half-time of the assumed exponential decrease in blood FDG concentration (40, 50 or 60 min) had little influence on these percentages, although, as would be expected, the percentages were slightly higher for faster half-times. The remaining FDG is un-phosphorylated, exchanging with blood FDG. These percentages increased at later times post-injection but did not approach 100% until several hours post-injection.

Whilst there has been much interest in measuring FDG clearance in several tissues for many years, there has been less interest in the physiological significance of tissue FDG distribution volume. Yet, as shown here, *V*(0) is critical in determining the proportion of FDG in a tissue that is actually phosphorylated. Several studies have reported how the SUVs of various tissues change between imaging at 60 min and delayed imaging, with some tissues showing an increase and others, notably the liver and spleen, showing a decrease in SUV [33–37]. The magnitudes and directions of the changes are determined by Ki/*V*(0). Thus, when Ki/*V*(0) is higher than *β*, there is an increase in SUV and vice versa when Ki/*V*(0) is lower than *β*. The issue of early versus delayed imaging is likely to increase in importance with the development of new whole-body PET machines such as UltraPET [38].

In the measurement, by computer modelling, of the transport constants in Fig. 1, previous workers have stressed the importance of considering the liver’s dual blood supply [1–3, 39]. There is general agreement, however, that Patlak-Rutland analysis, although less informative, is not influenced by the dual blood supply and is more robust than computer modelling for the measurement of Ki and *V*(0). Table 2 summarizes values of Ki and *V*(0) obtained by previous workers using computer modelling and Patlak-Rutland analysis. It can be seen from this Table that in humans, there is good agreement between modelling and Patlak-Rutland analysis [2] and that our mean value of Ki/*V*(0) for the liver, based on Patlak-Rutland analysis, is similar to previously published values in humans based on both modelling and Patlak-Rutland analysis [1, 2]. In anaesthetised pigs, Ki based on modelling was somewhat higher than human values [3]. Patlak-Rutland analysis, however, gave values of Ki/*V*(0) in line with other studies and our own values [1–3]. We can conclude therefore that our estimate of Ki/*V*(0) for the liver is reliable. We could find no corresponding literature values for the spleen.

**Table 2:** Hepatic Ki and *V*(0) determined by previous workers [1–3] using computer modelling and Patlak-Rutland graphical analysis

	Modelling			Graphical analysis		
	Ki	*V*(0)	Ki/*V*(0)	Ki	*V*(0)	Ki/*V*(0)
Choi et al. [1]	0.0043	0.88^a^	0.0049	ND	ND	ND
Iozzo et al. [2]	0.0029	0.81	0.0036	0.0023	0.84^b^	0.0029^b^
Munk et al. [3]^c^	0.0078	0.95	0.0082	0.0037	1.05	0.0035

Ki and *V*(0) based on modelling have been calculated using the equations linking Ki and *V*(0) to the transport constants (e.g. Ki = *K*_1_ × *k*_3_/[*k*_2_ + *k*_3_] and *V*(0) = *K*_1_/[*k*_2_ + *k*_3_])

Ki, ml/min/ml; *V*(0), ml/ml. Note that our mean value of Ki/V(0) was 0.0036 ml/min/ml

*ND* not done

FA – fat-adjusted

^a^ml/g

^b^Values from single illustrated Patlak-Rutland plot (mean of group values not given)

^c^Anaesthetized pigs

The spleen SUV was found to be less than the liver SUV even though it had a higher Ki/*V*(0). As shown in the Appendix, this is the result of a lower distribution volume in the spleen. Considering the relative values of hepatic and splenic SUV_max_ and Ki/*V*(0), hepatic *V*(0) can be calculated to be 1.6-fold higher than splenic *V*(0) (see Appendix).

SUV depends on the total tissue FDG concentration (FDG plus FDG6P), which partly explains the weak correlations we found between SUV indices and Ki/*V*(0) for both the liver and spleen. Interestingly, the strongest correlations were found with SUL indices, which support the use of LBM for calculating SUV [40]. The poor correlations of hepatic SUV_mean_ indices with Ki/*V*(0) is explained by the physical effect of hepatic fat on the FDG signal—a ‘fat-diluting’ effect that has previously been described to affect SUV_max_ less than SUV_mean_ [32]. Making the described adjustment for hepatic fat gave SUV_mean_ a stronger correlation with Ki/*V*(0) compared with the corresponding unadjusted values (approximately doubling the correlation coefficients for both SUW_mean_ and SUL_mean_; Table 1). With respect to the spleen, correlations were similar for SUV_max_ and SUV_mean_ indices because fat is not an issue in the spleen. Interestingly, the strongest correlation with Ki/*V*(0) was seen when splenic SUV was divided by hepatic SUV. Others have previously shown that division of tissue SUV by blood pool SUV makes it a better surrogate of clearance than SUV alone [41, 42]. As shown here, hepatic SUV is largely a blood pool SUV, explaining this finding.

A potential limitation of Patlak-Rutland analysis is the influence of glucose-6-phosphatase (*k*_4_), which if active would invalidate the requirement for a single transport pathway. Previous workers in this field, however, also recorded linear hepatic Patlak-Rutkand gradients, suggesting that de-phosphorylation is very slow [1–3]. In any event, we avoided a longer acquisition period in order to minimize a possible influence of de-phosphorylation on the gradient.

Study limitations include firstly the recruitment of patients with co-existing morbidity. Secondly, hepatic fat distribution is heterogeneous [43], so measurement of CT density in a single ROI for the hepatic fat adjustment procedure may be misleading. Moreover, CT is not regarded as the gold standard imaging technique for quantifying steatosis, but probably instead MRI and MR spectroscopy [44]. The duration of our dynamic acquisition may be considered limited but longer acquisition periods risk patient movement artefacts, especially in patient participants rather than motivated normal volunteers [1, 2, 4], as well as a possible influence of de-phosphorylation, so 30 min seemed reasonable. Others used 40 [2, 4] or 60 min [1]. A 2-min mixing time of FDG throughout the hepatic distribution volume may seem brief but is consistent with previously reported values of *K*_1_ and *k*_2_, which respectively ranged from 0.01 to 0.015 and 0.013 to 0.016 s^−1^ [1–3], and which therefore give an equilibration rate constant of 0.023–0.031 s^−1^. This gives a time to 95% equilibration of FDG between compartments 1 and 2 of 97–130 s. Munk et al. also found from rapid early sampling that equilibration was achieved within a few minutes [3]. We used abdominal aorta for arterial input but others have validated the abdominal aorta for Patlak-Rutland analysis [28, 45], including for the liver [45].

## Conclusions

In conclusion, tissue FDG6P concentration depends on FDG clearance *per unit total volume* but the FDG6P/FDG concentration ratio depends on tissue clearance *per unit distribution volume*. Because the majority of FDG is un-phosphorylated, SUV in both the liver and spleen largely reflects blood pool activity, especially the liver, explaining the poor correlations between SUV indices and Ki/*V*(0). If SUV is to be used as a measure of FDG clearance and metabolic activity, then LBM is the preferred whole-body metric with which to calculate it.

## Appendix

Equation 9 can be re-written, replacing FDG6 + FDG with SUV, for both liver (L) and spleen (S).

A1 $$ \frac{{\mathrm{SUV}}_S}{{\mathrm{SUV}}_L} = \frac{\left( V(0). A(t).{\left[\mathrm{Ki}/ V(0)\right]}_S\left)+ V{(0)}_S. C\right( t\right)}{\left( V(0). A(t).{\left[\mathrm{Ki}/ V(0)\right]}_L\left)+ V{(0)}_L. C\right( t\right)} $$

Note that administered activity and whole-body metric cancel out when the two SUVs are expressed as a ratio, which then becomes the ratio of the total FDG concentrations in the two organs.

Therefore, re-arranging and dividing numerator and denominator in Eq. A1 by *C*(*t*)

A2 $$ \frac{{\mathrm{V}(0)}_L}{{\mathrm{V}(0)}_S} = \frac{{\mathrm{SUV}}_L\times \left[ A(t)/ C(t).\right]{\left[\mathrm{Ki}/ V(0)\right]}_S + 1\Big)}{{\mathrm{SUV}}_S\times \left[ A(t)/ C(t)\right].{\left[\mathrm{Ki}/ V(0)\right]}_L + 1\Big)} $$

For disappearance exponential half-times of 40, 50 and 60 min, *A*(*t*)/*C*(*t*) is 105, 94 and 87 min, respectively. So for SUV_L_/SUV_S_ of 1.36 (see the “Results” section), the corresponding values of *V*(0)_L_/*V*(0)_S_ would be 1.61, 1.59 and 1.58.
